# Analysis of Cell and Molecular Phenotypes of a Highly Replicated Longevity-Associated FOXO3 Variant in Older Adults

**DOI:** 10.1093/geroni/igab046.1418

**Published:** 2021-12-17

**Authors:** Philip Davy, Randi Chen, Richard Allsopp, Bradley Willcox

**Affiliations:** 1 Kuakini Medical Center, Honolulu, Hawaii, United States; 2 Kuakini Medical Center, Kuakini Medical Center/Honolulu, Hawaii, United States

## Abstract

Aging demographics in the US, and other industrialized nations, are resulting in rapidly increasing health care costs from age-related diseases. New therapeutic interventions to extend healthspan in older adults requires understanding connections between basic aging biology and human longevity factors. Using clinical samples from the Kuakini Honolulu Heart Program (HHP) and their Offspring, we are examining potential links between molecular and cellular mechanisms of aging and the longevity associated FOXO3 genotype (carriage of SNP rs2802292 “G” allele). Telomere dynamics in leucocytes (LTL) have shown strong correlation with multiple lifestyle and health factors. We previously demonstrated a significant protective relation between FOXO3 longevity genotype and LTL in a cross-sectional study. Now we are assessing a longitudinal relation, at three time points over 20+ years, in older men. We are also exploring stem cell frequency and differentiation capacity in neurological and peripheral blood samples to assess FOXO3 genotype and human cell dynamics.

